# Phase-change metasurface slows down light

**DOI:** 10.1038/s41377-021-00637-z

**Published:** 2021-09-19

**Authors:** Din-Ping Tsai

**Affiliations:** Department of Electrical Engineering, City University of Hong Kong, Kowloon, Hong Kong China

**Keywords:** Metamaterials, Optical techniques

*Light: Advanced Manufacturing* 2, 19 (2021)

10.37188/lam.2021.019

All-dielectric metasurface analogue of electromagnetically induced transparency (EIT) is highly desirable for developing compact and low-loss nanophotonic devices, such as dispersion-tunable slow-light meta devices. However, it remains challenging to realize dynamic control of EIT in all-dielectric metasurfaces in the near-infrared region. To this end, researchers at the Dalian University of Technology in China have demonstrated active tuning of EIT-featuring Mie resonances in an all-dielectric metasurface based on patterned germanium antimony telluride (GST), a phase-changing material whose optical response differs significantly between the amorphous and crystalline phases. Experimentally, they have achieved a spectral tuning range of 360 nm and a relative modulation contrast of 80% at the EIT resonance wavelength by laser-induced phase switching of GST. Surprisingly, the extreme dispersion associated with the EIT resonance results in the group velocity of a near-infrared light beam 335 times slower than that in a vacuum. We anticipate that this optically tunable metasurface device can find its applications in a broad field, and the generic design approach can be extended to more comprehensive optical frequencies by using novel phase-changing materials.

